# Design and Implementation of the Amenah Early Marriage Pilot Intervention Among Syrian Refugees in Lebanon

**DOI:** 10.9745/GHSP-D-21-00079

**Published:** 2022-02-28

**Authors:** Maia Sieverding, Dima Bteddini, Rima Mourtada, Lama Al Ayoubi, Ola Hassan, Aya Ahmad, Jocelyn DeJong, Sawsan Abdulrahim

**Affiliations:** a American University of Beirut, Beirut, Lebanon.

## Abstract

Implementing and evaluating interventions in humanitarian settings in low- and middle-income countries presents unique challenges that are little addressed in the implementation literature. We document the process of developing, implementing, and evaluating the Amenah pilot intervention that aimed to mitigate the drivers of early marriage in a Syrian refugee community in Lebanon. Adolescent girls’ vulnerability to early marriage increases following displacement due to poverty, insecurity, and school disruptions. We delineate how, as a local research team, we triangulated evidence from the international literature and formative community research to make informed decisions during the intervention’s design and implementation phases. The pilot was delivered to 203 Syrian refugee schoolgirls aged 11–14 years during the 2017–2018 academic year. It consisted of 16 structured, interactive sessions with girls and a set of facilitated meetings with the girls’ mothers, both of which were implemented by trained female community workers from the Syrian refugee community. Process evaluation results showed that sociodemographic factors predicted attendance among mothers, but relationships with peers in the intervention were the only significant predictor of attendance among girls. The primary outcomes of the pilot were attitudinal measures related to education and marriage. Attitudes toward education were highly positive at baseline and did not change over the course of the intervention. There were no significant changes in girls’ ideal age at marriage. Among girls aged 13 and older at endline, the mean self-reported expected age at marriage increased slightly from 20.2 to 20.8 years (*P*<.05). Our results also suggest that girls may adjust their expected age at marriage downward as they become older and if they drop out of school. We reflect in the discussion on some of the challenges encountered and lessons learned for the benefit of researchers intending to conduct community-based interventions in displacement settings.

## Abstract (precis)

We document the design, implementation, and evaluation of an early marriage intervention among Syrian refugee adolescents in Lebanon and describe the adaptations made to address a range of factors related to the vulnerability and mobility of the refugee population.

## Teaser

**Key Findings:**

- The attitudes of refugee adolescent girls toward education were overwhelmingly positive at baseline, such that no change was detected at endline.
- Girls’ positive expectations about their education contrasted with their views on marriage; as they grew older, girls increasingly adjusted their age at marriage expectations to the normative patterns in their community.
- There was considerable discordance between girls’ attitudes/ideals and the circumstances of their lives.
- The quality of peer relationships between girls was the only factor that predicted their attendance in the Amenah project.

**Key Implications:**

- Interventions in displacement settings should involve refugees in the design and delivery of projects that serve their community.
- Project managers should invest in efforts to engage parents in displacement settings, particularly fathers, in interventions that focus on their children.
- Incorporating peer networks and “safe spaces” should be included as critical components of intervention projects with refugee adolescent girls.
- Ending early marriage among refugee adolescent girls cannot be achieved without mitigating structural constraints in displacement and enhancing children’s access to education.

## INTRODUCTION

The global increase in forced displacement has triggered substantial growth in public health research among refugees and internally displaced populations, motivated by the need to address poor health outcomes among these vulnerable groups. Most public health research with displaced populations, however, has been descriptive with limited attention to intervention approaches. This gap is particularly grave in low- and middle-income countries (LMICs), where there is a dearth in the quantity and quality of health interventions in humanitarian settings.^1^ Although several studies have documented the challenges of intervention research with refugees resettled in high-income countries,^2^^,^^3^ the literature is sparse in addressing the unique aspects of implementing and evaluating interventions with refugees in LMICs, and specifically in the Arab region, which hosts one of the largest refugee populations worldwide.

Lebanon hosts a large number of Syrian refugees who arrived in successive waves following the civil war that began in 2011. As of November 2021, there were 844,056 Syrian refugees registered with the office of the United Nations High Commissioner for Refugees in Lebanon, of whom 39.0% resided in the Eastern Governorate (Bekaa) and 54.3% were under age 18.^4^ This large population of refugee children and adolescents is highly vulnerable to poor socioeconomic and health outcomes. Although Lebanon committed to including Syrian refugee children in the public education system, adopting a condensed afternoon shift for them,^5^^,^^6^ the official education policy for Syrian refugees was not uniformly implemented and full integration into formal schools has faced numerous challenges.^5^^–^^7^ Data indicate that Syrian children in Lebanon show alarmingly low rates of school enrollment, particularly in Bekaa where the primary school enrollment rate stood at 55% in 2019.^8^

Low rates of school enrollment, in addition to insecurity and poverty, contribute to girls’ vulnerability to early marriage—defined as any formal or informal union where at least 1 of the parties involved is under 18 years of age^9^—during humanitarian crises.^10^^,^^11^ There is no rigorous evidence on trends in early marriage among Syrian refugees in Lebanon due to a lack of nationally representative data that can be compared with early marriage rates in pre-conflict Syria in a manner that accounts for the selectiveness of refugees, who are not representative of the national Syrian population pre-conflict. Nevertheless, several qualitative studies and reports by international humanitarian organizations have advanced the argument that the practice has increased as a result of displacement.^12^^–^^15^ A recent review of early marriage studies among Syrian refugees in Lebanon and Jordan also identified key themes, including financial challenges, fear of harassment, sexual violence, and sociocultural factors such as family honor and societal norms, that are argued to contribute to increased early marriage rates.^16^ Although most studies included in the review acknowledge that early marriage existed as a practice in Syria before the war, the authors suggest that new dynamics around early marriage have also emerged due to forced displacement.^16^ Thus, while it is unclear to what extent the rate of early marriage has changed among Syrian refugees in Lebanon, the literature consistently points to new drivers of the practice in the context of displacement.

Early marriage is both an outcome of and a contributor to socioeconomic and gender inequalities. The Sustainable Development Goal on gender equality (SDG-5) calls for the elimination of child, early, and forced marriage as harmful traditional practices that negatively impact girls in LMICs. Under conditions of insecurity and sudden loss of livelihood, refugee families may view the practice as a form of protection for adolescent girls.^10^^,^^11^ Yet early marriage is associated with school dropout, limited access to reproductive health services including contraception, and early pregnancy, which increases the risk of maternal mortality and other poor reproductive health outcomes.^11^ Given the many negative health and social consequences of early marriage and its purported increase in displacement settings, humanitarian organizations have called for developing and implementing programs to accelerate ending this harmful practice.

In this article, we document the process of developing, implementing, and evaluating a pilot intervention, Amenah, that aimed to mitigate the drivers of early marriage in a Syrian refugee community in the Bekaa region of Lebanon. Although early marriage among Syrian refugees has received attention from scholars and international organizations, to our knowledge there is no published literature about early marriage interventions that have been implemented among this population in Lebanon. Amenah—Arabic for girls/women who are secure and protected—pilot intervention was a multicomponent, community-based intervention implemented in 2017–2018 by a multidisciplinary research team in the Faculty of Health Sciences at the American University of Beirut, Lebanon. The pilot built on existing evidence on preventing early marriage in nonconflict settings and introduced additional components that addressed drivers of early marriage under conditions of displacement.

The pilot built on existing evidence on preventing early marriage in nonconflict settings and introduced additional intervention components that addressed drivers of early marriage under conditions of displacement.

## DEVELOPMENT OF THE AMENAH PILOT INTERVENTION

The Amenah pilot intervention was developed using a combination of a review of early marriage interventions implemented in nondisplacement settings, results drawn from previous studies conducted by the research team, and additional formative work in the community.

### Generating Quantitative Evidence on Early Marriage

In 2016, the research team conducted a survey on early marriage and its social determinants among Syrian refugees in 3 towns in Bekaa. The survey results revealed that 24.6% of girls aged 15–17 years were married. The survey also demonstrated inverse patterns regarding education and marriage. School enrollment rates were low among girls in the study area, with only 61.8% of girls aged 9–14 years and 20.3% of girls 15–17 years enrolled in formal or informal school. The Figure shows that school enrollment hovered around 70% for girls aged 9–12 years but then began to decline considerably starting at age 13 years. Marriage rates began to increase at age 15 years, by which point only one-third of girls were still in school.

**FIGURE:**
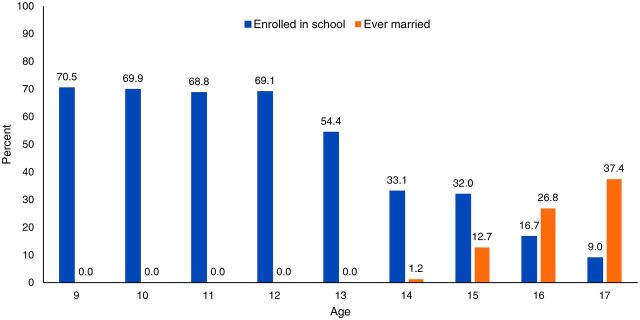
School Enrollment and Marriage Rates by Age Among Syrian Refugee Girls in Bekaa, Lebanon

**Figure:**
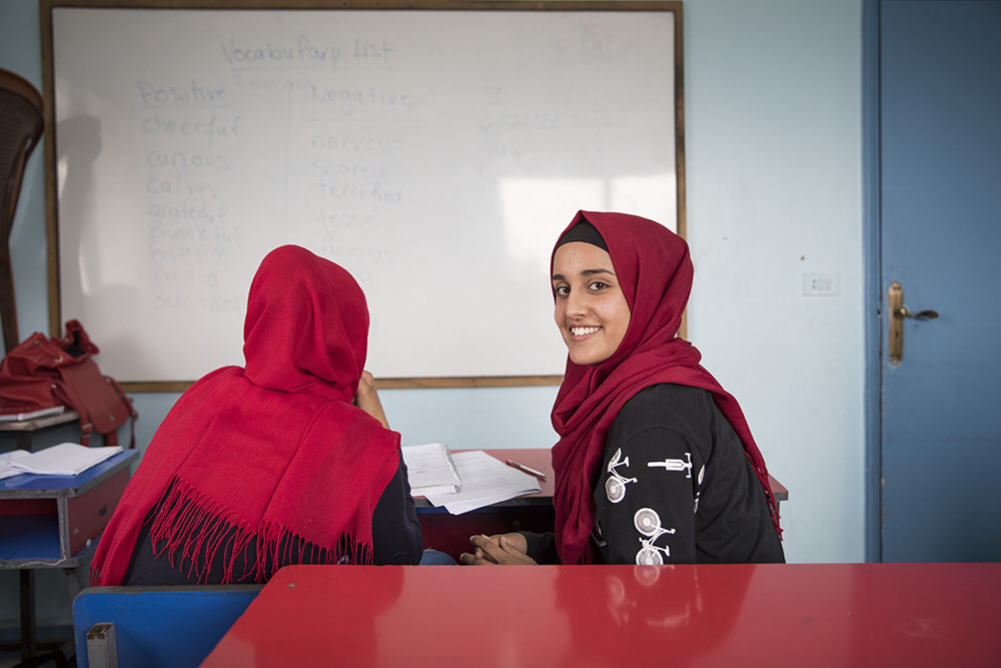
Adolescent girls attending an Amenah session in Lebanon. ©2019 Catalina Martin-Chico

The prevalence of early marriage among the study sample was considerably higher than the rate reported in preconflict Syria in 2009, where 11.6% of girls aged 15–19 years were married at the time of the survey and 17.3% of women aged 20–24 years women had married before age 18.^17^ However, as other studies have shown,^18^ Syrians displaced into neighboring countries are not representative of the preconflict Syrian population but are a select sociodemographic group. For example, most Syrian refugees captured in the 2016 prevalence study (95.5%) originated from 8 of the 14 Syrian governorates, reported having a rural origin (82.3%), and were from disadvantaged social classes as measured by parental education. The prevalence study therefore could not establish whether early marriage had increased among the refugee population as a result of displacement, due to the unavailability of robust data to compare marriage rates pre- and postdisplacement. Nevertheless, its findings concluded that the practice is prevalent in the study area. In light of this evidence, research team members decided to design and implement an intervention study to mitigate the drivers of early marriage.

The prevalence of early marriage among the study sample was considerably higher than the rate reported in preconflict Syria in 2009.

### Selecting the Girls’ Age Group and Intervention Site

The research team used evidence from the prevalence study to select the specific population of girls to focus on and the intervention site. In response to the trends displayed in the Figure, it was decided to intervene with girls aged 11–14 years who are at high risk of school dropout and early marriage. Moreover, the 11–14 years age range is when most girls typically enter puberty and, in the context of displacement, begin to experience mobility restrictions. The research team recognized that a large percentage of girls in this age range in the study area were out of school and that girls who do not attend school are at a higher risk of marriage. However, the decision to focus the Amenah pilot intervention on girls in school was based on evidence that keeping girls in school for as long as possible delays marriage,^19^ as well as practical considerations related to the ease of recruiting girls through schools compared to community venues for the initial pilot study.

The decision to implement the pilot intervention in 1 of the 3 towns included in the prevalence study was also based on empirical evidence. In disaggregating the prevalence study data, 1 town stood out in that school enrollment was higher among girls aged 9–12 years as compared to the 2 other towns (more than 80% of girls in this age group attended school) but enrollment dropped precipitously when girls were aged between 13 and 14 years. The results seemed to indicate that school attendance for young girls in this town was normative but dropped at age 13 years as a result of family- or school-related factors that the intervention could address.

### Developing the Conceptual Framework

A formative research phase aimed to inform the conceptual approach to the intervention and tailor it to the context of Syrian refugees in Lebanon. This phase encompassed 2 components: (1) review the evidence on what works to mitigate the drivers of early marriage in LMICs, with a focus on interventions in humanitarian settings; and (2) conduct a series of community meetings to discuss the intervention with Syrian refugees, particularly mothers of adolescent girls and other stakeholders representing humanitarian organizations, municipalities, and schools.

The team reviewed several systematic reviews that evaluated the rigor and impact of early marriage interventions and summarized the most effective approaches.^19^^–^^21^ The reviews revealed that high-quality, impactful interventions comprised multiple components and incorporated an empowerment approach either solely or in conjunction with other approaches. However, none of the interventions identified were implemented among a displaced, migrant, or refugee population. It thus became apparent that any conceptual framework we employed needed to be adapted to the context of displacement and the added vulnerabilities this context imposes on adolescent girls and their families.

We used the conceptual framework developed by Marcus and Page^22^ that lays out the main drivers of early marriage in LMICs in relationship to interventions to address these drivers (Table 1). According to the conceptual framework, these intervention approaches have the intended outcomes of changing norms around early marriage, strengthening girls’ bargaining power, increasing school enrollment rates, reducing economic incentives for early marriage, and more effective enforcement of early marriage laws. These outcomes in turn lead to reduced incidence of early marriage.^22^

**TABLE 1.** The Main Drivers of and Points of Intervention for Early Marriage According to the Marcus and Page^22^ Conceptual Framework

**Driver (Marcus and Page)**	**Intervention Approaches (Marcus and Page)**	**Amenah Intervention Component**
Sociocultural norms that favor early marriage	Provide information to girls and community members on consequences of early marriage through community-level dialogue and awareness campaigns	Girls’ sessions Mothers’ and fathers’ sessions
	Life-skills education including on legal rights and risks of early marriage provided in girl-only safe spaces	
Lack of knowledge of the law and consequences of early marriage	Provide information; life-skills education (as above)	Girls’ sessions Mothers’ and fathers’ sessions
Girls’ relative powerlessness	Support girls through income earning capacity and skills training; life-skills education	Girls’ sessions (for life skills education only)
Poverty and vulnerability	Strengthen family livelihoods; cash incentives for educational attendance	Not addressed
Lack of educational opportunities	Provide formal and nonformal education opportunities; cash incentives for educational attendance	English support
Weak law enforcement	Strengthen law enforcement	Not addressed

The research team acknowledged that some intervention elements proposed in the framework were impossible to implement (e.g., cash incentives) given the unavailability of resources and the anti-integrationist policy context in Lebanon. As such, the team decided to focus on intervention components that were feasible to implement during a short-term pilot study, particularly life-skills education for girls and addressing norms at the level of girls and their families. Moreover, although the framework proposed addressing girls’ empowerment through supporting their economic activities to earn incomes, we were concerned about this approach in a context where child labor is common. Therefore, the team decided to focus the Amenah intervention on empowerment through school retention and girls’ right to education.

The second component of the formative research involved meeting with community members and other stakeholders (Supplement Table). Families have a well-recognized role in decisions around early marriage and have been incorporated in numerous interventions to address the practice.^19^ Our formative research focused on mothers because in the Syrian refugee context they typically know more about their daughters than do fathers, and mothers play an important structural role of protector of the girl. Of note, the group meetings held with mothers revealed that they had limited knowledge of education policies for refugees in Lebanon and that, while they valued girls’ education, economic and security challenges forced them to accept early marriage as a form of protection for their daughters. Thus, the title of the project, Amenah, was selected to reflect the mothers’ desires to protect their daughters and the researchers’ proposition that adolescent girls deserve to be protected without having to marry early.

In focus-group discussion, mother’s revealed that, while they valued girls’ education, economic and security challenges forced them to accept early marriage as a form of protection for their daughters.

### Developing the Intervention Content and Evaluation Tools

Before implementation, the research team developed the intervention content and evaluation tools. As a pilot, the Amenah intervention used a 1-group pretest/post-test design that entailed an assessment of outcomes at baseline, implementation of the intervention, and assessment at endline, as well as process evaluation. Due to feasibility considerations, we did not include a comparison group in the pilot. The content of the intervention and assessment tools for the outcome evaluation were built in tandem; the assessment tools were designed to capture change between baseline and endline on core intervention components.

#### Intervention Content

The final Amenah pilot intervention consisted of 16 interactive sessions with girls and a set of facilitated meetings with the girls’ mothers and fathers (Table 1). The girls’ curriculum was developed by reviewing curricula from early marriage interventions that were previously implemented in nondisplacement settings with some similarities to Syria and Lebanon in terms of the sociocultural context, including Egypt,^23^ Bangladesh,^24^ and Ethiopia.^25^ We also took some curriculum activities from a previous intervention implemented with Palestinian refugee youth in Lebanon due to the similarities in the context and structural constraints faced by Syrian refugees.^26^ The selected activities were modified for the Syrian refugee context based on insights from the formative research as well as discussions with Syrian refugee mothers.

The curriculum was grouped into 5 units (the Supplement shows the full outline): communication and problem-solving; 2 units on life skills, including assertive communication and self-confidence; a unit on health, including puberty and menstruation; and a unit on gender and human rights that included the content on early marriage. Each session included objectives, instructions to the facilitator, and interactive activities. The activities stressed skill building and were designed with a focus on gender equality, encouraging girls to examine their perceptions. Sessions were delivered in a group setting, with each group holding between 12 and 17 girls.

Because of the academic challenges Syrian girls face in Lebanese public schools, particularly the fact that math and science are taught in English or French, it was decided to supplement the Amenah sessions with English language support sessions. The original idea of incorporating English language support came from the girls’ parents who, concerned that the Amenah sessions were not academic, requested including a component to help their daughters in their studies. Accordingly, the research team recruited volunteer university students to provide structured English language support. Because participants were schoolgirls, the Amenah sessions were scheduled during weekends, and the average time of the session was 2 hours, plus 1 hour of English support.

A set of 8 sessions for mothers were designed to complement the girls’ component and to provide a forum for discussion around adolescence and early marriage but with a stronger focus on gendered norms and mother-daughter communication. Initially, the intervention included 4 sessions for fathers that had similar objectives, given the importance of fathers for decision making around marriage. However, the fathers’ sessions were halted due to low attendance, a point we return to in the discussion.

#### Evaluation Tools

The research team developed several process and outcome evaluation instruments to monitor the intervention and capture key indicators at baseline and endline (Table 2).

**TABLE 2.** Summary of Data Collection Instruments and Procedures for Amenah Early Marriage Pilot Intervention Among Syrian Refugees in Lebanon

	**Instrument**	**Key Topics Covered**	**Administered By**	**Timing of Data Collection**	**Location of Data Collection**
Outcome Evaluation	Household questionnaire	Sociodemographics of household members; asset index	Community workers	Baseline/endline	Family home
	Girls’ questionnaire	School experiences and attitudes; gender role attitudes; attitudes toward education and early marriage; experience of puberty; mother-daughter communication; experience and satisfaction with Amenah (endline only)	Female university students	Baseline/endline	Partner NGO (MAPs)
	Mothers’ questionnaire	Gender role attitudes; attitudes toward education and early marriage; mother-daughter communication; experience and satisfaction with Amenah (endline only)	Community workers	Baseline/endline	Family home
	Fathers’ questionnaire	Gender role attitudes; attitudes toward education and early marriage	Community workers	Baseline/endline	Family home
Process Evaluation	Session observation form	Objectives achieved; activities implemented as planned; notes on session delivery	Community workers; external evaluators	Directly after each session	Partner NGO (MAPs)
	Attendance records	Session attendance of mothers and girls	Community workers	During each session	Partner NGO (MAPs)
	Mothers’ focus group discussion	Perceptions of Amenah; effect of Amenah on relationship with daughter	Study team	7 months after intervention	Partner NGO (MAPs)

Abbreviations: MAPs, Multi-Aid Programs; NGO, nongovernmental organization.

### Ethics Approval

Ethical approval for the study was obtained from the American University of Beirut Institutional Review Board. Approval was also obtained from the Lebanese Ministry of Education and Higher Education to recruit participants through schools. While waiting to obtain approvals, the research team established a partnership with, Multi-Aid Programs (https://www.linkedin.com/company/multi-aid-programs/), a local humanitarian organization that provides health, relief, and informal education services to Syrian refugees. Multi-Aid Programs is well known and well-respected in the Syrian community; its safe and easily accessible premises served as an ideal site for implementing the intervention sessions.

## RECRUITMENT OF COMMUNITY WORKERS AND PARTICIPANTS

### Recruiting and Training Community Workers

After careful consideration, the research team opted to hire and train Syrian female community workers (CWs), who have cultural knowledge of their community, to implement the intervention sessions rather than outside facilitators. A total of 14 adult women CWs, all of whom had a minimum ninth-grade education, were selected following an extensive recruitment and training process that spanned over 2 months. Some of the CWs had themselves married early or had daughters who married at young ages, which they expressed as a motivation to work on the intervention.

Because CWs were expected to conduct sessions with girls as well as facilitate sessions with mothers, the research team felt that engaging adult women in implementation would enhance the acceptability of the project by parents. Young and unmarried women would have been perceived by parents as not mature enough to relay information about health and early marriage. The research team decided to compensate the CWs financially for their work rather than expect them to volunteer their time given that they faced the same legal and economic constraints as participating families.

CWs underwent a 3-week training designed to achieve the objectives outlined in the Box. Despite the intensity and rigor of the training, it was perceived as a first step toward preparing the CWs; continuous training was integrated throughout the project. Every fourth week during the implementation phase, the CWs reconvened and provided feedback on the last 2–3 sessions they delivered (with girls and mothers) and received training on the content and delivery of the 2–3 upcoming sessions. The CWs also received 2 booster trainings by a gender empowerment expert and other mini skills-building sessions on an as-needed basis.

BOX **Objectives of Community Workers’ Training**

*Information*

Describe:

- Intervention components
- Roles and responsibilities of community workers
- Adolescent health and early marriage
- Resources available through local and international nongovernmental organizations

*Skills*

Demonstrate competence in:

- Session facilitation
- Use of tablets for data collection
- Survey techniques

*Group cohesion*

- Build a collegial and collaborative relationships between community workers
- Establish a common language around gender equity and girls’ rights

### Participant Recruitment and Consent Process

After obtaining IRB approval, the research and implementation teams (CWs and a community research assistant) embarked on recruiting and enrolling participants into the intervention study in sequential steps (Table 3). The team decided not to select girls based on their school grade because Syrian refugee children in Lebanon are often enrolled in a grade that is lower than the one expected for their age due to lost years of education during displacement. Eligibility was therefore determined by girls’ birthdates. Recruitment also took into consideration the importance of sensitizing parents to the intervention before beginning the formal consent process.

**TABLE 3.** Steps Taken to Recruit Participants in Amenah Early Marriage Pilot Intervention

**Step**	**Conducted by**	**Location**
Obtain lists of the names and birthdates of Syrian female students from the 4 partner schools	Research team in coordination with schools	Schools
Send letter about the study to parents of all girls aged 11–14 years, informing them about the study and inviting them to attend a meeting on school premises	Research team in coordination with schools	Schools
Hold a meeting in each school to describe the study and obtain preliminary consent from attending parents to be visited at home; record parents’ contact information	Research team and community workers, in coordination with schools	Schools
Conduct home visits to obtain the consent of parents to enroll their daughter in the pilot intervention	Community workers	Home visits
Obtain assent from girls to participate in Amenah	Research team	Community partner (MAPs) premises

Abbreviation: MAPs, Multi-Aid Programs.

The team encountered several challenges during the process of recruiting girl participants. Although refugee parents were used to providing information for needs assessments conducted by humanitarian organizations, they were not used to the concept of informed consent in research. Some parents who did not have legal documentation in Lebanon were eager to enroll their daughter in the intervention but expressed discomfort with the idea of signing the consent form. Moreover, some parents raised concerns about a clause in the consent form that girls who display poor mental health will be referred to professional services; as the girls’ baseline instrument included a mental health assessment tool, referral of girls who exhibited poor mental health was a requirement of the researchers’ institution’s ethics board. These challenges did not impact recruitment as CWs explained to parents the academic institution’s research ethics requirements and assured them that the research team will not refer their daughters to mental health services without notifying them and obtaining their consent. Moreover, despite their hesitation to sign the consent form, parents were eager to enroll their daughters in the intervention given the limited opportunities available for girls in that displacement context to engage in extracurricular activities in safe spaces.

## PILOT EVALUATION RESULTS

We used the combination of data sources (Table 2) to evaluate the Amenah pilot in terms of processes and outcomes. The primary outcomes of Amenah were increased school retention and later ages at marriage. Since it was not feasible to measure these long-term outcomes in the context of the pilot, we instead focused the outcome evaluation on attitudinal measures related to education and marriage, including girls’ self-reported expected age at marriage. Although the use of attitudinal outcomes is common in early marriage interventions due to the limited timeframe of most program evaluations,^27^ this approach makes the (strong) assumption that changed attitudes will ultimately lead to changed behaviors.

We focused the outcome evaluation on attitudinal measures related to education and marriage, including girls’ self-reported expected age at marriage.

Since the intervention was a pilot being conducted in a challenging and changing environment, feasibility and acceptability were also important considerations. Therefore, we examined several domains of Linnan and Steckler’s^28^ process evaluation framework. These include: (1) reach, or the degree to which the focus population was engaged by the program, assessed through the number and characteristics of participating girls and their households; (2) fidelity, or the degree to which the intervention was carried out as planned, assessed through the observation of sessions; and (3) dose received, or participant engagement in the program, assessed through mothers’ and girls’ satisfaction with and perceptions of the program, and predictors of mothers’ and girls’ attendance. Several measures were taken to reduce courtesy bias in responses to satisfaction measures; girls were surveyed by university students with no other involvement in the intervention, and, at endline, mothers and fathers were surveyed by a CW other than the one who was responsible for delivering their daughters’ sessions.

### Process Evaluation

#### Program Reach: Characteristics of Participating Girls and Their Households

At the time of recruitment, there were 340 eligible Syrian girls enrolled in the 4 participating schools; 270 (79%) of the girls’ parents attended one of the meetings introducing the project. A total of 210 girls and their households (62% of eligible girls) participated in the baseline survey, of whom 203 girls attended any of the Amenah sessions. However, there was some attrition both during the intervention and between the end of the sessions in September 2018 and the endline survey in November–December 2018. We have endline data for 178 participating girls (85% of girls enrolled at baseline), all but 2 of whom attended at least 5 Amenah sessions. We focus our analysis of the pilot results on this panel sample that constitute 52% of all eligible girls included in the intervention. Girls who were lost to follow-up were not significantly different from those who were retained in the study either on individual characteristics or residence in an informal tented settlement (ITS); parents’ characteristics could not be assessed because this information was taken from the endline survey.

As expected, given that recruitment was based on school enrollment, participants and their households were socioeconomically advantaged relative to the Syrian refugee population in Lebanon overall. The percentage of families in our study who resided in tented settlements (20%; Table 4) was lower than for Syrian refugees in the Bekaa (48%).^8^ A substantial number of girls had parents with some level of education, with 52% of mothers and 53% of fathers having preparatory education or higher (62% among fathers with valid data). These numbers are higher than national estimates for Syrian refugees in Lebanon, among whom only 23% of men and 21% of women above age 15 have completed ninth grade or higher.^29^ The percentage of working fathers in our sample (68%) is also higher than that of working Syrian refugee men on a national level (46%).^8^

**TABLE 4.** Characteristics of Amenah Early Marriage Pilot Intervention Participants and Their Households

	No. (%)
**Household characteristics**	
Residence in ITS	
No	143 (80)
Yes	35 (20)
**Mothers' characteristics**	
Mother age, years	
25–29	6 (3)
30–34	47 (26)
35–39	56 (31)
40–44	34 (19)
45+	24 (13)
Missing	11 (6)
Mother education	
Primary or less	75 (42)
Preparatory or higher	92 (52)
Missing	11 (6)
Mother's age at marriage, years	
<16	31 (17)
16–17	51 (29)
18–19	37 (21)
20–24	42 (24)
25+	14 (8)
Missing	3 (2)
**Fathers' characteristics**	
Father education	
Primary or less	57 (32)
Preparatory or higher	95 (53)
Missing	26 (15)
Father employment	
Not employed	32 (18)
Working but not regularly	76 (43)
Working regularly	44 (25)
Missing	26 (15)
Father UNHCR registration status	
Registered	133 (75)
Not registered	19 (11)
Missing	26 (15)
**Girls' characteristics**	
Age at baseline, years	
11	28 (16)
12	63 (35)
13	54 (30)
14	33 (19)
School attended at baseline	
Public elementary school	43 (24)
Public middle school	44 (25)
NGO school 1	33 (19)
NGO school 2	58 (33)
Grade level at baseline	
3	6 (3)
4	32 (18)
5	33 (19)
6	63 (35)
7	31 (17)
8	13 (7)
Grade level compared to age	
In expected grade level or higher	75 (42)
1–2 grade levels below expected	93 (52)
3 or more grade levels below expected	10 (6)
Total	178 (100)

Abbreviations: NGO, nongovernmental organization; UNHCR, United Nations High Commissioner for Refugees.

^a^N=150 due to missing data on fathers’ age.

Although participants were socioeconomically (relatively) advantaged, early marriage was common among the girls’ mothers, with 46% having married below age 18 and an additional 21% at age 18 or 19. This is consistent with literature that argues that early marriage among Syrian refugees in Lebanon is in part a continuation of traditions from Syria.^12^ Fewer than half (42%) of girls were attending the expected grade level for their age, whereas the rest were 1 or more grade levels behind. Correspondingly, girls aged 11–14 years were enrolled in a wide range of grades, from grades 3 to 8.

#### Program Fidelity

High percentages of both CWs (98%) and external observers (90%) reported that at least 75% of the session objectives were met. However, considerably lower percentages (50% of CWs and 40% of observers) reported that the session activities were fully implemented. Key challenges that were noted in terms of session implementation included: the large number of girls in some groups; certain activities that needed more time than allotted (e.g., drawing and role play); chaos and noise; having to deal with different “styles” such as engaging girls who were shy; having to respond to unexpected topics raised during the sessions; and poor time management or repetition of materials leading to not all activities being completed.

Both CWs (98%) and external observers (90%) reported that at least 75% of the session objectives were met, but only 50% of CWs and 40% of observers reported that the session activities were fully implemented.

#### Dose Received: Satisfaction and Attendance

Both mothers’ and girls’ reported satisfaction with Amenah was very high (Table 5), which likely reflects a courtesy bias in the survey responses, despite our efforts to reduce it, as well as actual perceptions of the program. Due to the very high overall ratings, in most cases questions that were originally asked on a 5-point Likert scale ranging from “very bad” to “very good” were recoded for analysis to compare “very good” responses to all others.

**TABLE 5.** Mothers’ and Girls’ Satisfaction With the Amenah Program

	**No. (%)**
**Mothers' satisfaction (N=172)**	
In general, how would you rate Amenah?	
Neutral to good	69 (40)
Very good	102 (60)
How would you rate your relationship with the community worker?	
Neutral to good	42 (24)
Very good	130 (76)
How would you rate your relationship with the other mothers?^a^	
Neutral	25 (21)
Very good/good	92 (79)
**Girls' satisfaction (N=175)**	
In general, how would you rate Amenah?	
Neutral to good	24 (14)
Very good	151 (86)
The Amenah facilitator gave me important information.	
Agree somewhat	9 (5)
Strongly agree	166 (95)
I can share my private feelings and problems with the Amenah facilitator.	
I do not agree at all	31 (18)
Agree somewhat	70 (40)
Strongly agree	74 (42)
Did you make friends during Amenah?	
No	36 (21)
Yes	138 (79)
How would you rate your relationship with the other girls in Amenah?	
Neutral/bad	44 (25)
Good	50 (29)
Very good	81 (46)

a N=117 due to missing data.

The social relationships among participants and between the participants and the CWs were expected to be a key aspect of program acceptability and efficacy. Respondents were again very positive about these aspects of the program. However, whereas nearly all girls strongly agreed that they received important information from the CW, only 42% strongly agreed that they could share their private feelings and problems with her, suggesting that their affective relationships with the CWs were more variable. This may be in part because the CWs were in many cases close to the age of the girls’ mothers and maintained close contact with them; as such, they were perceived by the girls to play more of a parental role of protector rather than a peer or confidant. The large majority of girls also said that they made friends in Amenah, but the reported quality of their relationships with the other girls varied.

Findings from follow-up focus group discussions with mothers similarly indicated very positive views about the project. Mothers were particularly positive about the sessions on puberty and menstruation. They acknowledged that they had difficulty addressing the subject with their daughters and stated that the girls’ session on menstruation took this burden off their shoulders.

*Honestly, I was happy [that my daughter learned about menstruation during an Amenah session], because the girl is too shy to talk to her mother… You relieved us from the matter.*

Mothers also stated that their daughters became more open with them after participating in Amenah and gained self-confidence and assertiveness, highlighting that these changes improved their relationships with their daughters.

*[My daughter] became more open and talks to me more. As for me, I became more able to understand her. I mean she used to be shy, but now she became courageous.*

*[…] My daughter became more mature. [In the past,] if she was catcalled on the street, she used to cry. Now she became more mature and able to defend herself. And her character became stronger.*

Given the likely bias in self-reported satisfaction with the program, we examined mothers’ and girls’ attendance in the sessions as an important indication not only of the amount of program content they received but also the acceptability and perceived value of Amenah to participants. Mothers’ attendance was considerably more variable than that of girls. Of the 175 mothers in the panel data, 67% attended any session and, among these, the average number of sessions attended was 3 of a total of 8. The attendance outcome for mothers was therefore categorized as attending at least 1 session versus not attending any sessions. Among girls, by contrast, 80% attended 10 or more sessions of a total of 16. The mean number of sessions attended by girls was 12. High attendance among girls was therefore categorized as near-complete attendance (14–16 sessions; 34%) versus attending 13 sessions or fewer (66%).

We examined mothers’ and girls’ attendance in the sessions as an important indication not only of the amount of program content they received but also the acceptability and perceived value of Amenah to participants.

We assessed a variety of factors that may have influenced attendance, including geographic barriers, such as residence in the ITS (transportation was provided for girls’ but not mothers’ sessions); characteristics of the mother and father that may influence prioritization of the program over other activities, such as their education and employment status; the age of the girl; the school, in case certain schools were more or less encouraging of participation in Amenah or had an academic load that may have competed with participation; and satisfaction with the program and relationships with other participants. In Table 6, we examine each of these potential predictors of higher program attendance using bivariate logistic regression.

**TABLE 6.** Bivariate Predictors of Higher Attendance Among Mothers and Girls

** **	**Unadjusted Odds Ratio** **(Standard Error)**	
	Mother Attendance (1+ sessions)	Girl Attendance (>66% of sessions)
Residence (Ref =outside ITS)		
Inside ITS	1.30 (0.579-2.938)	1.41 (0.656-3.012)
Mother education^a^ (Ref =primary or less)		
Preparatory or higher	0.57 (0.297-1.084)	1.07 (0.560-2.031)
Missing	—	0.75 (0.183-3.075)
Mother age	1.09^b^ (1.024-1.161)	1.00 (0.947-1.062)
Father education (Ref =primary or less)		
Preparatory or higher	1.20 (0.597-2.397)	1.31 (0.648-2.656)
Missing	0.50 (0.179-1.397)	1.25 (0.464-3.343)
Father employment^b^ (Ref =not working)		
Work but not regularly	1.78 (0.703-4.495)	0.95 (0.391-2.333)
Work regularly	0.34 (0.132-0.895)	1.52 (0.584-3.974)
Missing	0.42 (0.133-1.310)	1.16 (0.387-3.501)
Father age	1.09^b^ (1.016-1.165)	1.00 (0.947-1.066)
Girl age, years (Ref =11)		
12	1.14 (0.431-3.026)	0.67 (0.267-1.662)
13	0.73 (0.281-1.907)	0.67 (0.261-1.704)
14	1.09 (0.368-3.229)	0.50 (0.171-1.459)
Girl current school (Ref =public primary)		
Public middle school	0.54 (0.205-1.435)	1.57 (0.657-3.770)
NGO school 1	0.89 (0.306-2.603)	0.90 (0.338-2.397)
NGO school 2	0.50 (0.199-1.231)	0.86 (0.366-2.014)
Girl attended more than 66% of sessions (Ref =no)	1.71 (0.835-3.518)	
Very good overall rating of Amenah^a^ (Ref =neutral to good)	1.02 (0.531-1.975)	1.05 (0.422-2.617)
Very good relationship with CW (Ref =neutral to good)	1.05 (0.500-2.194)	
Made friends during Amenah (Ref =no)		1.07 (0.491-2.319)
Relationship with other girls in Amenah^a^ (Ref =very good)		
Good		0.39 (0.175-0.869)
Neutral/bad		0.72 (0.333-1.535)
CW provided important information (Ref =agree a little)		1.05 (0.252-4.338)
Agree a lot		
Can share private feelings with CW (Ref =do not agree)		
Agree a little		1.24 (0.507-3.039)
Agree a lot		1.01 (0.411-2.471)
Observations	175	178

Abbreviations: CW, community worker; ITS, informal tented settlement; NGO, nongovernmental organization.

a *P*<.1.

b *P*<.01.

Different factors predicted attendance among mothers versus girls. All the factors that predicted higher mothers’ attendance were sociodemographic. Mothers with a higher level of education (at least preparatory) had lower odds of attending any sessions, which may indicate that they were less likely to see value in or prioritize Amenah relative to those with lower education. Mothers in households where the father worked irregularly were more likely to attend than mothers in households where the father did not work at all. By contrast, mothers in households where the father worked regularly were less likely to attend sessions. This may be because women whose husbands were at work had less mobility to leave the home, potentially due to the presence of other children. Both older mothers’ ages and fathers’ ages were associated with greater odds of attendance, which may also be related to younger parents having small children at home. None of the satisfaction variables predicted attendance among mothers. Among girls, by contrast, the only variable that significantly predicted high attendance was having a very good (as opposed to good or neutral/bad) relationship with other girls in Amenah.

Another important factor about the perceived value of Amenah that emerged from the qualitative data was the mothers’ views on the English language support sessions. Despite being reminded of the aims and different components of Amenah, mothers recalled and discussed the English support sessions extensively, stating that their daughters benefited a lot from them. This emphasis on the English support is likely due to the difficulties that many Syrian students face in the Lebanese school system due to their lack of English-language proficiency. Similarly, in a mental health intervention with Palestinian refugee youth in Lebanon, parents focused on academic aspects of the intervention and discounted the importance of activities designed to build life skills.^30^ While academic support is thus an important incentive that may attract and retain adolescents from refugee households in health interventions, approaches to helping parents understand the value of life skills or empowerment-oriented activities are needed.

### Outcome Evaluation

#### Girls’ Attitudes Toward Education and Early Marriage

Girls’ attitudes toward education were overwhelmingly positive at baseline, such that no change was detected at endline. For example, 92% of girls at baseline and 90% at endline said that the ideal level of education for a girl to reach is university or above. In terms of their expectations for their education, 80% of girls at both baseline and endline said that they expected to achieve university or higher. Of the 178 girls in the panel, 10 (6%) left school between the 2017–2018 and 2018–2019 academic years. Interestingly, even among these 10 girls, 6 reported at endline that their expectation for their education was to complete university. These findings suggest a mix of social desirability and discordance between girls’ expectations and the reality of their situations.

These very positive expectations about their education also contrasted somewhat with girls’ views on marriage. The mean reported ideal age at marriage for a woman was 20.1 years at baseline and 20.4 years at endline (Table 7). The fairly young ideal age at marriage, while older than age 18 years, emphasizes the discordance with girls’ views on education, as typical ages at finishing university would be age 22 or 23 years. There was no significant change in girls’ views on ideal age at marriage at endline, whether for the sample overall, by educational enrollment at endline or by age.

**TABLE 7.** Baseline-Endline Comparison of Age at Marriage Outcomes

	**Baseline**	**Endline **
**Ideal age at marriage**		
Total	20.1	20.4
School status at endline		
Enrolled	20.2	20.5
Not enrolled^a^	19.1	18.8
Age at endline, years		
13	20.0	20.1
14	20.2	20.8
15–16	19.5	20.4
**Own expected age at marriage (girls aged 13+ years at endline)**		
Total	20.2	20.8^b^
School status at endline		
Enrolled	20.3	20.9^b^
Not enrolled^a^	19.0	18.5
Age at endline, years		
13	20.3	20.6
14	20.6	21.4
15–16	19.1	20.1^c^

a Only 6 girls were not enrolled in school at endline and had complete data on the outcome.

b P<.05, based on paired t-test.

c P<.1, based on paired t-test.

There was no significant change in girls’ views on ideal age at marriage at endline, whether for the sample overall, by educational enrollment at endline or by age.

Girls aged 13 years and older at endline were also asked about their own expected age at marriage. The average age increased slightly for the participants overall, from 20.2 years at baseline to 20.8 years at endline (*P*<.05). These results were driven by the majority of girls who remained in school. The number of girls who had dropped out of school by endline and had complete responses on expected age at marriage was very small (N=6). Nevertheless, it is interesting to note that among these girls their expected age at marriage was younger than average at baseline and declined further by endline. Additional research is needed on this topic, but school dropout may be related to a process of expectations adjustment in which girls begin to expect to marry sooner as their prospects for continuing education decrease. This process may be reinforcing, if girls with lower expected ages at marriage consequently begin to decrease their school attachment.

We also hypothesized that girls’ current age may be related to their expected age at marriage. If girls’ expectations increasingly adjust to the normative patterns of their communities as they grow older, their actual age and expected age at marriage should begin to converge toward ages 17–18 years. Our data provide some limited evidence for this dynamic; both ideal and expected ages at marriage were slightly lower among girls aged 15–16 years. However, girls aged 15–16 years were the only age group for which a marginally statistically significant increase in own expected age at marriage was seen, from 19.1 at baseline to 20.1 at endline (*P*<.10). Keeping in mind the small sample size for this group, it is nevertheless possible that the pilot intervention had more impact on older girls who were closer to typical ages at marriage in the study community than girls who were still younger than age 14. This may be because older girls were more likely to think of marriage as an event that is near in time to their own age and were, therefore, more attentive to messages about early marriage.

## DISCUSSION

We documented the process of developing, implementing, and evaluating an early marriage pilot intervention among Syrian refugee adolescent girls in Lebanon. Our experience with the Amenah intervention can provide lessons learned for other interventions with displaced populations, particularly those that adopt a community-based approach or are implemented in contexts characterized by substantial structural barriers to improving refugee health. Our article thus fills an important gap in the literature, that of limited documentation of interventions carried out in refugee settings in LMICs generally and the Arab region in particular.

### Lessons Learned

The Amenah pilot experience highlights the importance of involving refugees in the design and delivery of interventions that serve their community. The female Syrian CWs who implemented Amenah served as a link between the community and the research team and established a trusting relationship with the girls’ parents. The involvement of members of marginalized communities in public health research enhances its relevance and contributes to reducing social inequities.^31^ The importance of involving refugees in delivering health services in humanitarian settings has also been noted in other contexts.^32^ In displacement settings, however, researchers ought to recognize how economic and legal factors affect implementers from excluded communities who genuinely commit to the research project but whose contributions are constrained by multiple and competing demands. The Syrian CWs who implemented the Amenah intervention shared the same socioeconomic background and displacement experience as the girls’ families. While this affinity to the community enhanced cultural understanding and parental acceptance, it meant that CWs faced the same social, economic, and legal hardships that all Syrian refugees in Lebanon face and, as women, endured patriarchy in their own community.

CWs played an important role related to the positionality of the researcher vis-à-vis both the study community and implementers. Systematic evidence demonstrates that most research on health and the Syrian conflict has been led by international investigators who are unfamiliar with the social and political context of the Arab region, raising questions not only about the validity of the findings but also about ethics and power.^33^ Situated at a Lebanese university, we were positioned as local researchers who understood the social context and made decisions based on both scientific evidence and cultural knowledge. However, following the formative phase, we had limited if any interactions with girl participants, parents, or the Syrian community at large. CWs were the link that connected us to the community, and almost everything we learned about the day-to-day workings of the intervention sessions came through information they relayed during regular meetings. Despite our positionality as local researchers, the implementation of the intervention would not have been successful without maintaining a close relationship with CWs who realized the challenges of countering early marriage under the economically constrained conditions of displacement. This further serves to emphasize the need to involve displaced populations in the delivery of health interventions.

The critical role of parents in effective interventions to address the drivers of early marriage, given parents’ role in decision making for their daughters, has been well demonstrated.^19^ Yet, the Amenah pilot suggests that more needs to be understood about how to engage parents in displacement settings in interventions that focus on their children. We were ultimately unable to engage fathers in the intervention, which is likely related to competing economic priorities among this displaced population as well as the perception that matters related to daughters are more the mother’s domain. Attendance among mothers was more variable and appeared to be affected by household-level factors. Although childcare services were provided in mothers’ sessions, transportation or transportation fees were not, which may have reduced the likelihood of attendance. Other researchers have also highlighted how structural inequalities that affect participation stymied the impact of community-based research in the highly inequitable context of Lebanon.^34^

The pilot suggests that more needs to be understood about how to engage parents in displacement settings in interventions that focus on their children.

Mothers’ focus on the English support sessions is also likely a reflection of the structural barriers that refugee children face in the Lebanese school system. While providing English lessons was important both for families’ commitment to the intervention and for directly supporting girls’ school retention, families’ focus on academic support may undermine their understanding of the components of the intervention that aim to empower girls or address norms around early marriage. It is important for interventions in this context to find ways to reinforce program content for parents while maintaining their engagement.

For girls themselves, our experience points to the value of “safe spaces” programs for adolescent girls that adopt the idea of peer support explicitly in their theories of change.^23^^,^^25^ The quality of their relationship with other girls in the program was the only factor that predicted girls’ attendance in Amenah, suggesting that peer networks are important for program attachment and potentially acceptance of content. Displacement may increase adolescent girls’ social isolation due to the disruption of peer networks from Syria and safety concerns in the host country, but the use of safe spaces approaches in nondisplacement settings^19^ also indicates that the importance of peers may be more general to girls of this age group. These peer dynamics are an element of Amenah and other interventions focused on adolescent girls that deserve further attention and development. For example, in the Amenah program expansion, the research team plans to use a peer educator model in which Syrian university students, who are closer to the girls’ ages and may thus be seen more as role models, deliver the intervention content to girls while the CWs deliver the mothers’ sessions.

Our preliminary results suggested that there may be considerable discordance between girls’ attitudes/ideals and the circumstances of their lives. Educational attachment was high, as expressed through girls’ attitudes, and desired ages at marriage were above secondary school age. Girls’ stated aspirations to achieve secondary or university education may be influenced by parents; 93% of mothers said that the ideal age of education for a girl was university, and 73% said that they expected their daughter to complete secondary or university education. Educational attainment among women in Syria had also increased considerably before the war. Yet despite the high value attached to education, we know that dropout rates increased considerably among this age group. These findings again point to the role of structural constraints in shaping the educational outcomes of Syrian refugee girls. The substantial shortcomings of Lebanon’s education policy for refugees,^5^^,^^7^ along with poverty and insecurity, lead families to prioritize girls’ security over their education despite preferences for the latter.^12^^,^^14^ Strengthened efforts to enroll and retain Syrian children in school are necessary to address this challenge. Cash transfers are an effective strategy to increase school enrollment and delay marriage of girls in other contexts.^35^ Although this approach was not feasible for Amenah to implement, it should be tested for Syrian refugees in Lebanon. Notably, given the displacement context, cash transfers would need to be accompanied by continued efforts to ensure that there are spaces for Syrian children in Lebanese public schools.

Our preliminary results suggested that there may be considerable discordance between girls’ attitudes/ideals and the circumstances of their lives.

### Limitations

In addition to the many challenges encountered in the field, there are several overarching limitations of the Amenah pilot study. Our focus on schoolgirls excluded a substantial percentage of girls in the study area from the pilot. We address this limitation in the expansion of Amenah that includes both in- and out-of-school adolescent girls. Our participants are also not representative of the population of in-school Syrian girls since joining the intervention was voluntary; we do not have the data to assess how our participants differed from eligible girls in the study area overall. Although the community-based model has important strengths, it also has drawbacks. These include some loss of rigor in terms of the consistency of implementation and data collection as a result of working with CWs who had not performed these functions before, as well as a likely increase in desirability bias in our evaluation data. We were also not able to include a comparison group in our evaluation due to feasibility considerations and were unable to measure impact on behavioral outcomes (school retention and early marriage) due to the short timeframe. Moreover, although we included a validated Arabic self-efficacy scale in the evaluation tools, we concluded that this was not well understood by girls and therefore do not have measures of girls’ agency. Our results in terms of program outcomes are therefore only indicative.

Perhaps the most important limitation of Amenah is related to the limits of an empowerment-based approach when that approach cannot address the structural legal and economic constraints on refugee households. These constraints not only shape the focus population’s ability and willingness to engage with the intervention but are also a key driver of early marriage itself. Our experience with the Amenah pilot thus highlights the importance of policy advocacy alongside community-based intervention approaches. In the absence of policies to support the economic integration of refugees and a more concerted effort on the part of the formal school system to keep Syrian children in school, interventions such as ours can only have limited effect. This is of particular importance in the context of the ongoing coronavirus disease (COVID-19) pandemic; from March 2020 through March 2021, Syrian refugees only had 3 months of school, whether online or in-person, which is likely to have contributed to permanent school dropout and increases in early marriage rates. In terms of community-based practice with refugee communities, our key lessons learned for future interventions is that rigorous training of and meaningful engagement with refugee implementers of intervention components in displacement settings are critical to success. Engagement not only promotes trust but also prepares researchers to better understand the intersecting impact of poverty and patriarchy on the lives of refugee girls and women, and the findings of their research.

Our experience with the Amenah pilot thus highlights the importance of policy advocacy alongside community-based intervention approaches.

## Supplementary Material

21-00079-Abdulrahim-Supplement.pdf

## Notes

**Peer Reviewed**
