# Supplementary material for: Design and Implementation of the Amenah Early Marriage Pilot Intervention Among Syrian Refugees in Lebanon
Source: Glob Health Sci Pract. 2022 Feb 28;10(1):e2100079. doi: 10.9745/GHSP-D-21-00079 (PMC8885338; doi:10.9745/GHSP-D-21-00079)
Supplement: 21-00079-Abdulrahim-Supplement.pdf [file 21-00079-Abdulrahim-Supplement.pdf]

Supplement to: Sieverding M, Bteddini D, Mourtada R, et al. Design and implementation of the Amenah early marriage pilot intervention among Syrian refugees in Lebanon. *Glob Health Sci Pract*. 2022;10(1):2100079. https://doi.org/10.9745/GHSP-D-21-00079

Supplement Table. Stakeholder meetings held during the formative research

Stakeholder(s)	Purpose
8 group meetings with Syrian refugee mothers of adolescent girls	• To discuss specific elements of the Marcus and Page framework<br>• To learn about the social context in which Syrian refugees live and challenges they encounter
One-on-one meetings with staff from local and international humanitarian organizations	• To learn about existing programs/activities related to child protection, adolescent health, and early marriage
One-on-one meetings with four school principals and the mayor of the town	• To gain the school principals' and Mayor's buy-in and request their staff's support during the recruitment phase

Supplement: Units of the *Amenah* Girls' Curriculum

Unit One: Introduction
Session 1. Introducing the Program/Ground Rules/Collective Decision-Making
Session 2. The School (problems and solutions)
Session 3. The Family (problems and solutions)
Session 4. The Community (problems and solutions)

Unit Two: Communication Skills
Session 5. Verbal and Non-Verbal Communication and Styles of Communication
Session 6. Practicing Assertive Communication
Session 7. Maintaining Friendships and Social Support

Unit Three: Psychosocial Life Skills
Session 8. Self-Awareness and Self-Confidence
Session 9. Social Problem Solving / Stress Management / Managing Our Emotions
Session 10. Resisting Peer Pressure

Unit Four: Health
Session 11. Healthy Lifestyle
Session 12. Changes in Adolescence

Unit Five: Human Rights
Session 13. Human Rights and Gender
Session 14. Gender Role Analysis
Session 15. Early Marriage I
Session 16. Early Marriage II and closing
